# Thyroid Storm Triggered by Partial Hydatidiform Mole: A Rare and Life-Threatening Complication

**DOI:** 10.1055/a-2626-9145

**Published:** 2025-06-18

**Authors:** Hailey Cox, Maggie Wong, Jena Preszler, Nikolina Docheva, Nauman Khurshid

**Affiliations:** 1 Department of Obstetrics and Gynecology, University of Toledo and Toledo ProMedica Hospital, Toledo, Ohio; 2 Department of Maternal-Fetal Medicine, Toledo ProMedica Hospital, Toledo, Ohio

**Keywords:** partial molar pregnancy, partial hydatidiform mole, thyroid storm, gestational trophoblastic disease, preeclampsia with severe features

## Abstract

A 26-year-old woman, gravida 1 para 0 at 14 weeks' 1-day gestation, presented with vaginal spotting and systemic symptoms, including palpitations, shortness of breath, heat intolerance, nausea, and vomiting for 2 weeks. Workup revealed maternal tachycardia, severe-range blood pressure, elevated beta human chorionic gonadotropin of 2,442,400 mIU/mL, suppressed thyroid stimulating hormone, and elevated T4, consistent with thyroid storm with possible preeclampsia with severe features. 1 A transvaginal ultrasound suggested a partial molar pregnancy; this was later confirmed by surgical pathology. This case highlights the rare yet serious complications of hydatidiform mole, in particular, a partial molar pregnancy, including thyroid storm and superimposed preeclampsia, emphasizing the importance of management at a tertiary care center with a multidisciplinary team to optimize maternal outcomes. 2 3

## Case Report

A 26-year-old woman, gravida 1 para 0 at 14 weeks' 1-day gestation (based on her last menstrual period and confirmed by an 8-week, 1-day ultrasound), presented to a community hospital emergency department with new-onset vaginal spotting. She had been experiencing intermittent heart palpitations, shortness of breath, night sweats, hot flashes, nausea, and vomiting for 2 weeks. Her medical, surgical, and family history were unremarkable. She was taking only a prenatal vitamin, had no known allergies, and denied the use of tobacco, alcohol, or illicit drugs. She had received regular prenatal care, including a normal first-trimester ultrasound.

On arrival at the outside hospital, her vital signs were as follows: heart rate = 170s beats/min, blood pressure = 146/63 mm Hg, respiratory rate = 18 breaths/min, temperature = 36.8°C, SpO2 = 99% on room air. Initial notable laboratory values were beta-human chorionic gonadotropin (β-hCG) = 2,442,400 mIU/mL, thyroid stimulating hormone < 0.02 mIU/mL, T4 >24.9 ug/dL, hemoglobin (Hgb)  = 9.8 g/dL. Other notable laboratory values included new-onset proteinuria with a urine protein–creatinine ratio of 0.34.

**Fig. 1:**
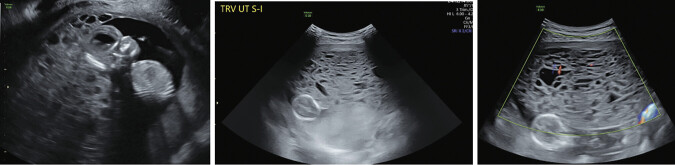
Transvaginal ultrasound of partial hydatidiform mole with classic “snowstorm” appearance.

**Fig. 2:**
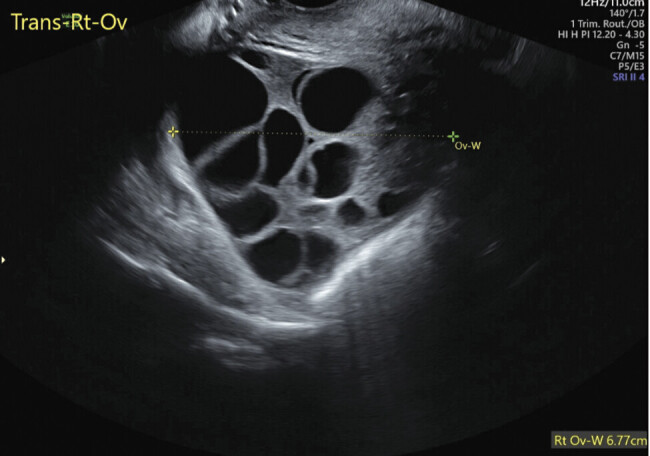
Transvaginal ultrasound of theca lutein cysts within ovary at the time of partial hydatidiform mole diagnosis.

A transvaginal ultrasound revealed a gestational sac with a fetal pole measuring 80 mm (14w0d), a fetal heart rate of 137 beats/min, posterior placenta with echogenicity suggesting a snowstorm appearance, most consistent with partial molar pregnancy ( Fig. 1 ). Theca lutein cysts were also noted bilaterally on full surveillance of the pelvis ( Fig. 2 ).

The patient was diagnosed with suspected partial molar pregnancy complicated by thyroid storm and hypertensive emergency. 4 5 6 7 She was transferred to a tertiary care center for management. She was given propranolol for heart rate control prior to transport.

**Fig. 3:**
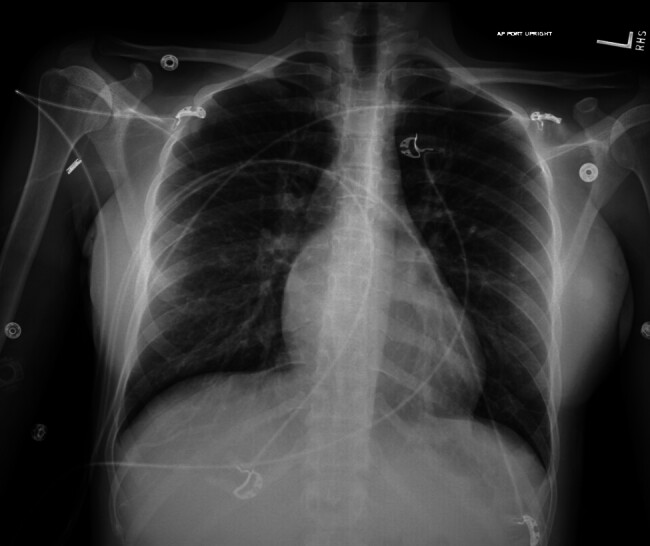
Negative chest X-ray at the time of partial hydatidiform mole diagnosis.

The patient received care from a multidisciplinary team, including obstetrics and gynecology, maternal–fetal medicine, critical care, endocrinology, and gynecologic oncology specialists. Her notable vital signs on arrival were a heart rate of 110 beats/min and blood pressure of 155/82 mm Hg. Her case was immediately discussed with maternal–fetal medicine and the critical care team. She was placed on continuous telemetry and pulse oximetry monitoring and initially had hourly blood pressure monitoring. A chest X-ray demonstrated no acute findings ( Fig. 3 ). Her electrocardiogram revealed sinus tachycardia and an echocardiogram showed a normal ejection fraction, mild-to-moderate mitral regurgitation, and probable patent foramen ovale.

The patient had multiple sustained severe-range blood pressures with a high suspicion for superimposed preeclampsia with severe features. She was started on intravenous (IV) magnesium for seizure prophylaxis. Her blood pressures were initially treated with IV antihypertensive agents followed by long-acting oral agents, including labetalol and nifedipine XL, which were slowly titrated up throughout her hospital stay. For the management of thyroid storm, she was started on propylthiouracil (PTU) 1,000 mg orally followed by 200 mg orally every 6 hours, hydrocortisone 100 mg IV every 8 hours for three doses, and potassium iodide 0.25 mL orally every 8 hours (2 hours following PTU).

Given the partial molar pregnancy, thyroid storm, and concern for preeclampsia with severe features, the decision was made to proceed with medical termination with dilation and evacuation due to the high risk of maternal morbidity and mortality. 4 Preoperative preparation for surgery included laminaria placement the night before and preparation for possible postpartum hemorrhage during the procedure.

Dilation and evacuation under ultrasound guidance were performed. She received 200 mg orally doxycycline for surgical prophylaxis. Her quantitative blood loss was 1,200 mL. She received TXA 1 g IV, misoprostol 1,000 µg rectally, and 2 units of packed red blood cells intraoperatively. Otherwise, the procedure was uncomplicated. Her Hgb remained stable at 8.7 g/dL and higher for the remainder of her admission. She was transferred to the SICU following the procedure for close monitoring. Endocrinology and gynecologic oncology were consulted.

She was continued on PTU 200 mg orally every 4 hours and hydrocortisone 100 mg IV every 8 hours for thyroid storm management along with labetalol 300 mg orally every 8 hours and nifedipine XL 30 mg orally every 12 hours for blood pressure management. Due to persistently elevated blood pressures, oral antihypertensive agents were titrated accordingly. Magnesium sulfate was discontinued after 24 hours.

**Fig. 4:**
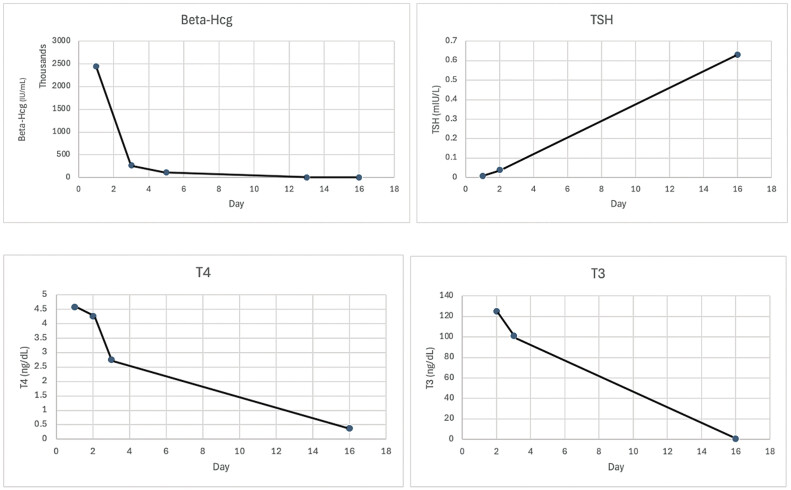
Trend of β-hCG and TSH profile before and after management of partial hydatidiform mole. β-hCG, beta human chorionic gonadotropin; TSH, thyroid stimulating hormone.

On postoperative day 1, the patient's laboratories was downtrending appropriately ( Fig. 4 ). IV hydrocortisone and orally PTU were discontinued and she was started on methimazole 15 mg daily. The patient was discharged on POD 3 once her tachycardia had resolved, blood pressures were < 150/< 100 mm Hg, and she was meeting all other postoperative milestones. She was discharged on methimazole 15 mg orally daily, labetalol 600 mg orally every 8 hours, and nifedipine XL 30 mg orally every 12 hours. Repeat β-hCG laboratories were being trended weekly until undetectable, then monthly for 6 months to monitor for progression to gestational trophoblastic neoplasia. 9 The pathology returned in 1 week and demonstrated hydropic villi tissue mixed with chorionic villi, decidua, and male fetus, consistent with partial hydatidiform mole. The patient received a Nexplanon for reliable contraception at her postoperative visit.

## Conclusion

This case highlights the complexity of a partial molar pregnancy and its rare, potentially fatal complications, including thyroid storm and superimposed preeclampsia, emphasizing the importance of early recognition and multidisciplinary team coordination to optimize maternal outcomes. 1 Similar to complete hydatidiform moles, partial molar pregnancies can manifest a thyroid storm if β-hCG levels are high enough. 8 Early intervention and expertise from a multidisciplinary team at a tertiary care center were crucial to the successful recovery of this patient. Treatment with antithyroid medications, corticosteroids, antihypertensive agents, and expedient surgical management was critical in optimizing outcomes. 7 Postoperative surveillance is essential, including β-hCG monitoring, effective contraception, blood pressure control, and appropriate follow-up care with specialists. 9 The clinical management of this case is summarized in Table 1 .

**Table 1:** Clinical management overview

Clinical focus	Actions and intervention
Initial evaluation	• Vital signs: heart rate, blood pressure, temperature, respiratory rate • Labs: β-hCG, TSH, T4, CBC, urine protein/creatinine • Imaging: detailed anatomy ultrasound, chest X-ray
Diagnosis	• Partial molar pregnancy • Thyroid storm • Preeclampsia with severe features
Available services	• Transfer to a tertiary center • Consults: ObGyn, Maternal–Fetal Medicine, SICU, Endocrinology, Gynecology Oncology
Medical management	Thyroid storm • PTU 1,000 mg orally loading dose, followed by 200 mg orally q6h • Hydrocortisone 100 mg IV q8h • Potassium iodide 0.25 mL orally q8h (2-h post-PTU) • Propranolol 20 mg orally as needed for tachycardia (HR > 100) Preeclampsia • Magnesium sulfate IV for seizure prophylaxis • IV and orally antihypertensives: labetalol, nifedipine, and hydralazine as needed
Surgical management	Dilation and evacuation Preop: laminaria, doxycycline Intraop: TXA, misoprostol, blood products
Postoperative care	• SICU monitoring • Continue antihypertensives and magnesium sulfate • Transition from PTU to methimazole 15 mg orally daily (continue methimazole outpatient) • Discontinue steroids
Surveillance and follow-up	• Weekly β-hCG until undetectable, then monthly ×6 mo • Blood pressure monitoring • Long-acting reversible contraception (e.g., Nexplanon) • Close follow-up with ObGyn and endocrinology

Abbreviation: β-hCG, beta human chorionic gonadotropin; CBC, complete blood count; HR, heart rate; IV, intravenous; Intraop, intraoperative; ObGyn, obstetrician–gynecologist; Preop, preoperative; PTU, propylthiouracil; TXA, tranexamic acid; SICU, surgical intensive care unit; TSH, thyroid stimulating hormone.
